# Attitudes and preferences of older adults and healthcare professionals regarding a multidisciplinary hybrid telehealth program for healthy aging

**DOI:** 10.3389/fpubh.2026.1777557

**Published:** 2026-03-17

**Authors:** Nesrine Koubaa, Mélanie Levasseur, Mylene Aubertin-Leheudre, Céline Verchère, Cinthia Corbin, Patrick Boissy

**Affiliations:** 1 Research Center on Aging, CIUSSS Estrie CHUS, Sherbrooke, QC, Canada; 2 Faculty of Medicine and Health Sciences, Université de Sherbrooke, Sherbrooke, QC, Canada; 3 Nanosystems Nanotechnologies Laboratory (CNRS 3463), Interdisciplinary Institute for Technological Innovation – 3IT, Sherbrooke, QC, Canada; 4 Faculty of Sciences, Department of Physical Activity Sciences, GRAPA, UQAM, Montreal, QC, Canada; 5 Centre de Recherche Universitaire de Gériatrie de Montréal (CRIUGM), Montreal, QC, Canada

**Keywords:** healthy aging, multidisciplinary program, qualitative study, telehealth, user-centered design

## Abstract

**Background:**

While aging, there is an increased risk of mobility decline, leading to deconditioning and reduced social participation in older adults. *PromoSanté* (short for “health promotion” in French) is a hybrid telehealth program currently under development that aims to combine in-person and remote occupational therapy, mental health and physical activity to support healthy aging among individuals with or at risk of deconditioning. Although promising, it is important to investigate the healthy aging challenges, older adults’ attitudes in the context of such programs, and the preferences of healthcare professionals to better inform both the development and the implementation process implementation.

**Objective:**

This study thus aimed to explore: (1) the challenges older adults encounter in achieving healthy aging, and (2) the attitudes and preferences of older adults and healthcare professionals regarding *PromoSanté*, including expectations for performance and effort, implementation factors to consider, and program design preferences, also to (3) identify and validate the main structure and forms of the user journey map of the program.

**Methods:**

A descriptive qualitative design was used, including semi-directed interviews with eight older adults and six healthcare professionals. Meetings with academic, clinical, and community partners informed discussions on program structure and participant journey.

**Results:**

Participants reported physical, mental and social vulnerabilities, in addition to transportation issues as challenges to healthy aging. *PromoSanté* was viewed as potentially beneficial for physical, mental, and social well-being. Healthcare professionals highlighted the digital format’s possibilities to improve accessibility and flexibility. A hybrid delivery model was preferred. Successful implementation requires adapting to older adults’ digital literacy and ensuring sufficient resources. A training session at program onset was recommended to reduce technology-related frustration and support engagement.

**Conclusion:**

Both older adults and healthcare professionals showed strong support for a multidisciplinary telehealth approach. Sustainable adoption depends on addressing individual and systemic factors.

## 1 Introduction

Deconditioning significantly limits mobility in older adults through declines in muscle strength, aerobic capacity, and balance (1), leading to reduced functional capacities and increased sedentary behavior and physical inactivity (2, 3). Older adults have been observed to spend an average of 8.5 h per day as sedentary (4, 5), which heightens risks of cardiovascular disease, falls, and cognitive decline (6, 7). Physical inactivity also impacts mental health and social engagement, contributing to loneliness and isolation (8–11). With an aging population (12, 13), preventing deconditioning is a public health priority. Promoting physical activity is key of healthy aging (6, 14). However, in-person physical activity programs face logistical barriers that hinder participation (15, 16).

The COVID-19 pandemic accelerated telehealth adoption to address access barriers in traditional care (17, 18). Telehealth is defined as healthcare delivered remotely via digital technologies (19). It includes synchronous methods like live video sessions with real-time feedback (20–22), and asynchronous options such as pre-recorded videos, offering greater flexibility for patients (23). Despite its benefits, telehealth can pose challenges for older adults. While increasingly using internet (24), some older adults report computer anxiety and concerns about privacy and security (23, 24). Limited digital literacy and experience with technology may cause frustration and reduce access (25–27). Additionally, visual, hearing, or motor impairments challenge use of telehealth tools (28). As a result, successful implementation may require additional training and support (29) compared to traditional methods.

Regardless of these challenges, telehealth is increasingly used for physical activity interventions among older adults and is generally perceived as feasible and well accepted (30). Studies report improvements in functional capacities, reduced sedentary and inactive behaviors, and enhanced quality of life following such interventions (31). However, effectiveness varies depending on delivery modalities and program features. While asynchronous formats (e.g., web-based programs) are common (30), synchronous methods like videoconferencing have been associated with better adherence and physical outcomes (32–35). A scoping review by Diaz et al. found attendance rates for synchronous programs ranged from 57% to 100%, with higher adherence in well-structured programs lasting over 12 weeks (36). Programs addressing access barriers, safety, and physical limitations through tailored support tend to achieve higher adherence. Interactive and socially supportive designs are especially effective in engaging older adults (36). Occupational therapy (OT) programs can enhance older adults’ engagement as it emphasizes meaningful daily routines, social support, and behavior change techniques (37). Interestingly, previous studies have shown promising results of some OT interventions delivered via telehealth, promoting functional capacities, active routines, and self-management among older adults (38–42). Merging physical activity with OT in a telehealth approach seems to offer a more holistic and valuable strategy for developing effective interventions of healthy aging in community-dwelling older adults.

In this context, a team comprising academic researchers specializing in physical activity, social participation and technological innovations, as well as community and clinical partners, was formed to collaborate on developing *PromoSanté* (short for “health promotion” in French), a telehealth program that aims to promote active lifestyle routines among older adults. *Promosanté* was built through a living lab approach (43) and intersectoral collaboration to ensure its relevance and feasibility. More specifically, *PromoSanté* integrates sessions of physical exercise, preventive OT modules (44), in addition to psychological support modules. Physical exercises component was inspired from the Move50+ program that was designed by a community center in Sherbrooke for individuals aged 50 and above offering recorded training program according to their fitness level (45). Preventive OCT modules were adapted to French from the Lifestyle Redesign® program, which aims to enhance social participation in older adults and promote fulfilling lives. The program includes various topics that help older adults achieve meaningful activities using presentation, dynamic exchanges and reflexives exercises (37). Overall the multidisciplinary content included in PromoSanté project is intended to prevent deconditioning among older adults by using an online platform. While understanding users’ perspectives prior to implementation is key to identifying factors that influence engagement and guiding program design (46), users’ attitudes is often assessed after prototypes are developed and implemented. Consequently, usability and acceptability of such programs can be limited. Furthermore, literature focuses on individual-level experiences, leaving a gap in understanding the organizational and other contextual challenges of implementation from the perspective of health care professionals (HCP) such as managers, practitioners and administrators (47). This study therefore explored the challenges older adults face to healthy aging, as well as their preferences and expectations—along with those of health care professionals—regarding the development and implementation of *PromoSanté*. Additionally, the study aimed to form and validate a user journey map to inform and optimize program design and delivery.

## 2 Materials and methods

### 2.1 Conceptual framework

To support the development of an inclusive and effective program, the *User-Centered Design* (UCD) approach was applied throughout the development of *PromoSanté*. Rooted in UCD principles (48), i.e., ensuring that the program is accessible, user-centered, and aligned with the needs and realities of both end users (older adults) and organizational users (HCP), the process involved users at every stage and unfolded over four phases: (1) exploring user expectations and preferences and mapping the participant journey, (2) designing and testing the program’s usability, (3) implementing the intervention, and (4) conducting ongoing evaluation and improvement. Engaging potential users—older adults, and HCP in the healthcare system such as providers and professionals involved in promoting healthy aging, the present study focused on the initial phase. Since it provides a comprehensive model for analyzing factors that influence the adoption of technology, especially in consumer contexts (49), the *Unified Theory of Acceptance and Use of Technology 2 (UTAUT2)* was also used. UTAUT2 has been widely validated for understanding technology acceptance among older adults (50), helping to identify key expectations and preferences of users, in addition to facilitators and barriers, to inform both the design and implementation of digital health interventions.

### 2.2 Study design

To generate in-depth, contextual insights into participants’ perspectives on *PromoSanté*, allowing for a nuanced understanding of their experiences and expectations, this study employed a descriptive qualitative design (51) with individual semi-directed interviews. The findings are intended to inform decision-making throughout the development and implementation of the program (52, 53). The study protocol was approved by the *CIUSSS de l’Estrie CHUS* research ethics committee (2022-4277).

### 2.3 Participants and recruitment

Participants (6 HCP 8 older adults) were recruited through a convenience sampling strategy from a community center and a hospital located in an urban region (Sherbrooke, Quebec, Canada). Both partner sites (community and hospital centers) invited individuals who met the following inclusion criteria to participate in the study: (1) being 65 years of age or older; (2) experiencing limitations in at least one activity of daily living (e.g., grocery shopping or housework); (3) residing in a conventional home or a private older adults’ residence; (4) having a good understanding of French; and (5) being able to navigate on internet. To ensure internal validity related to participants’ ability to understand the interview questions, older adults with severe cognitive or language impairments were excluded from the study. The research coordinator contacted people showing interest to participate and assessed inclusion criteria through a telephone eligibility screening. HCP affiliated to both partner sites were recruited through personalized emails and a snowball sampling approach, if they had at least 2 years of experience working with older adults and/or in delivering telehealth services. Older adults and HCP with relevant professional experience constitute information-rich participant groups that are closely concerned by the study topics. This specific sampling strategy would help enhance the depth of the data collected, and gather relevant insights to address the research questions of the study.

### 2.4 Data collection

Semi-directed interviews were conducted among each participant between July and August 2022. All were in-person interviews, except one conducted via Microsoft Teams. Prior to the interview, participants completed a demographic questionnaire. A semi-structured interview guide composed of open-ended questions was developed and validated by the research team members with diverse backgrounds (physical activity, social participation, gerontology, design and health technology implementation) to adequately cover relevant topics related to the research questions. Questions included in the guide were pretested with two patient partners. The primary interviewer wasn’t involved in participants’ clinical care and had no supervisory role over either older adults or HCP, limiting potential power imbalances during interviews. Participants were first investigated about the challenges older adults encounter in achieving healthy aging, and identifying their unmet needs. Thereafter, the interviewer provided an overview of *PromoSanté* program as a proposed program intended to address healthy aging challenges, particularly those related to physical and social limitations. The program was introduced as a multidisciplinary program focusing on healthy routines and tools, combining physical exercises, preventive OCT and psychological support. To ensure a concrete understanding of the proposed intervention, the interviewer illustrated some use cases for typical user scenarios (e.g., attending a virtual group physical exercise session, accessing community resources, and interacting virtually about aging related topics). The program description has also highlighted that virtual modalities will be supported by an online platform, a secure digital space, that will be developed to allow end users to access synchronous and asynchronous sessions. Inspired by key dimensions of the UTAUT2 framework, particularly performance expectancy (perceived usefulness), and effort expectancy (perceived ease of use) the questions focused on participants’ expectations and preferences regarding the program and its implementation. The interviews also included questions about the facilitators or barriers to the development and implementation of *PromoSanté*. When conducting interviews, the research team iteratively refined the interview guide to explore more deeply the emerging concepts. Furthermore, the interviewer engaged in reflexive memo-writing to note emerging impressions and contextual factors that may inform data analysis. Overall, interviews lasted between 30 and 45 min (median = 36 ± 3.5 min), were audio-recorded and transcribed for analysis. After the interviews, two co-design meetings with key partners (managers from community and hospital centers), were organized to discuss the interview results and decide *PromoSanté* implementation settings.

### 2.5 Data analysis

Interviews content were analyzed using a thematic approach as described by Miles and Huberman (54), including a mixed-method grid. The initial grid included principal themes that were identified basing on the study aims and the UTAUT 2 constructs, such as healthy aging challenges, performance expectancy, effort expectancy and facilitating conditions. Throughout the data collection, an inductive approach was adopted to capture sub-themes and relevant segments that were not represented within the UTAUT2 framework. Accordingly, the coding grid was iteratively refined to integrate new emergent themes identified within team discussions. Data analysis was conducted manually by the doctoral research and the project coordinator who hold formal training in qualitative methods and prior experience in conducting and analyzing interviews. To minimize bias and ensure that the themes accurately reflect participants’ thoughts, the collected data was coded independently to generated initial themes. Thereafter, discrepancies from this initial coding were discussed with the principal researcher of the study and disagreements were resolved by returning to the original transcripts, reflexive memo-notes and interactive interpretations until consensus was reached. The final synthesis of identified themes was illustrated using tables to clearly display each category and its related themes and sub-themes. The qualitative results were then presented during co-design meetings with different partners of the project. This step helped to identify key touchpoints between the older adults and HCP involved in the program during their participation in *PromoSanté* journey.

## 3 Results

During this study, recruitment continued until data saturation was considered as achieved, when the research team found that no new themes emerged in the final interviews and new verbatim confirmed the existing themes rather than expanding them. Overall, 8 older adults were recruited (7 women, median age = 72 ± 9 years old) with basic to higher education levels- (Table 1). Half of them lived alone and the majority reported having comorbidities and using different electronic devices to connect to the internet such as mobiles, laptops, and tablets. The sample also included 6 HCP (5 women, median age = 36.7 ± 16.8 years old) who held diverse professional positions and had none to, respectively, 2 or 25 years of experience with telehealth or working with older adults (Table 2). Results showed a variety in participants’ age, education level, living situations, health conditions among older adults, as well as in professional roles and years of experiences among HCP, which help capturing a range of perspectives across diverse contexts.

**Table 1:** Older adults’ characteristics.

Older adult (OA)	Gender	Age	Education level	Living situation	Number of comorbidities	Devices used to connect to the internet
OA 1	F	72	Secondary	With family	4	Tablet, laptop, mobile
OA 2	F	68	Secondary	With family	4	Laptop, mobile
OA 3	F	83	Higher	With family	1	Laptop, mobile
OA 4	F	79	Secondary	Alone	3	Tablet, mobile
OA 5	F	72	Higher	Alone	0	Tablet, laptop, mobile
OA 6	F	86	Secondary	Alone	4	Laptop
OA 7	F	66	Secondary	Alone	2	Laptop
OA 8	M	72	Basic	With family	2	Tablet

**Table 2:** HCP characteristics.

Participant	Gender	Age	Actual work position	Affiliation	Years of work experience with older adults	Years of work experience in telehealth
HCP 1	F	50	Chief of a rehabilitation department	Hospital	25	2
HCP 2	M	30	Physiotherapist	Hospital	7	2
HCP 3	F	40	Director of physical and entertainment activities	Community Center	12	0
HCP 4	F	30	Planning, programming and research officer	Hospital	0	2
HCP 5	F	55	Community support worker for older adults	Community Center	12	0
HCP 6	F	33	Volunteer coordinator	Community Center	14	0

### 3.1 Aim 1: Challenges to healthy aging

Older participants and HCP described similar struggles faced by older adults in their daily life with two main challenges to healthy aging: multidimensional situations of vulnerability, including physical, mental social dimensions, as well as transport barriers. While both themes were identified, vulnerability was more consistently emphasized by participants.

#### 3.1.1 Multidimensional situations of vulnerability of older adults

Older adults reported situations of vulnerability particularly through experiences of fatigue or loneliness, whereas HCP emphasized its broader consequences such as risks of falls, loss of appetite leading to malnutrition, and dehydration. These multifaceted situations of vulnerability were perceived as decreasing considerably the quality of life and hindering their daily, social, and instrumental activities.

##### 3.1.1.1 Physical

Physical vulnerability was broadly mentioned by both older adults and HCP as a factor leading to many restrictions in mobility, as shared by one older adult (OA): “*I have difficulty to walk because, after a few steps, I experience intense physical pain and shortness of breath. I sometimes choose not to go out specifically due to these persistent symptoms*.” (OA5). HCP (*n* = 3/6) described how older adults, notably those with balance disorders and chronic pain, are at risk of losing their autonomy. These limitations not only decrease mobility but also safety, thereby enhancing their risk of physical deconditioning, as reported by one participant: “*We usually treat cases of falls and balance problems, loss of autonomy in post-hospitalization deconditioning. For people at high risk of falling, going out is not safe.”* (HCP2).

##### 3.1.1.2 Mental and social

Older adults (*n* = 4/8) expressed difficulties when engaging in social interactions and making new relationships. They found it challenging to develop and maintain a social network where they can have meaningful exchanges and entertaining activities: “*I find it hard to have people with the same interests as me.”* (OA7). Social connection challenges were also explained by communication barriers, i.e., older adults (*n* = 2/8) revealed that they found struggles to structure their ideas, especially during group discussions. These issues sometime contribute to limit older adults’ motivation to participate socially, as highlighted by one participant: “*It could be an issue having difficulty expressing ideas and speaking in front of a group. A lack of vocabulary can be embarrassing. These could prevent older adults from participating or being comfortable in relationships.”* (OA4).

In the absence of peer support, many older adults experienced loneliness and anxiety, a mental vulnerability that can be exacerbated by the loss of close family members as mentioned by one participants: “*Since my husband left, most of the problem is an unwanted solitude… That I must tame, and it’s not easy… There’s always a part where you are alone; go to bed alone, get up alone, eat alone.”* (OA2). Older adults may face further mental vulnerability when witnessing health of their peers of the similar age rapidly declining until passing away. This may lead to emotional distress that can increase their anxiety about their future loss of autonomy and well-being and uncertainty about their resilience to health changes: “*A year after my husband’s death, 12 of our friends have died. We do not know if they will diagnose me with anything.”* (OA6).

From HCP perspective, loneliness and social deconditioning figure among factors that can affect interventions’ accessibility and effectiveness. They often face challenges not only to reach isolated older population, but also to keep them engaged and compliant to health interventions: “*We have people who are very, very isolated and who are more vulnerable… sometimes it’s harder to reach them, and, when we do reach them, sometimes it’s harder to keep them involved.”* (HCP3). According to some experiences of HCP (*n* = 2/6), social deconditioning can extend beyond psychological consequences, impacting fundamental health related behaviors such as hydration and nutrition. These effects compromise overall well-being and can lead to serious complications. For instance, HCP participants mentioned that the lockdown imposed broadly due to the COVID-19 pandemic had clearly shown how social restrictions could endamage older adults’ lives, as HCP1 mentioned: “*Even in the fifth wave of the pandemic, we had very few deaths related to covid. Deaths were mostly related to dehydration and eating problems because patients who had deconditioned socially ate much less.”*

#### 3.1.2 Transport barriers

Concerns about transportation accessibility and safety may further contribute to physical inactivity and social participation restrictions. Indeed, existing transport options may not be adapted to physical abilities of older adults, making their use difficult or even unsafe. Consequently, older adults are not comfortable to go outside and participate in community activities, especially in Quebec (Canada): “*People find it harder to get out in winter when they must shovel snow and take the car. For adapted transport, it’s a little more complicated. For the bus, in winter, they are afraid of falling.”* (HCP5). Also, an older adult affirmed: “*For some people, there must be a problem getting around to take part in the* var*ious activities.” (OA 6).*

### 3.2 Aim 2: Attitudes and preferences regarding *PromoSanté*

Participants’ attitudes were expressed through three main themes: their expectations from PromoSanté essentially in terms of performance and ease of use (Table 3), and factors influencing implementation (Table 4). Globally, participants believed that *PromoSanté* could present benefits that extend beyond health to include improvements in aging-care practices. Besides, participants perceived the ease of use of *PromoSanté*. According to the factors influencing implementation, HCP enumerated facilitators that can support the execution of such a program under real-world conditions and pointed out some barriers to consider.

**Table 3:** Participants’ expectations.

Performance expectations of the program		Effort expectations according to	
For older adults	For key actors	Older adults	Key actors
Multi-dimensional benefits Health self-management improvement Deconditioning prevention Easy lifestyle integration	Expanding the scope and accessibility of interventions Supporting clinicians’ practices Managing resources efficiently operable in real-world interventions’ contexts	Adapted physical intensity Time to learn and get familiar with technology Acceptable time commitment	Less physical and mental load Physical environment adaptation and equipment adjustment User-friendly interface

**Table 4:** Factors influencing implementation.

Facilitating conditions for the implementation	Barriers for the implementation	
	Individual barriers	Organizational barriers
Digital literacy improvements among older adults Co-design of the program components Training tools Organizational measures	Privacy concerns Challenges in adopting new Technologies by older adults Accessibility issues to online interventions	Challenges in integrating new work practices Strict cybersecurity standards

#### 3.2.1 Performance expectancy

Participants unanimously highlighted the numerous benefits expected from *PromoSanté* implementation. More specifically, older adults pointed to the anticipated benefits in terms of promoting health and well-being. HCP perceived that *PromoSanté* could improve clinical and organizational practices by enhancing accessibility to services and supporting more efficient resource management.

According to most older adults, multidisciplinary was recognized as the key asset that could significantly reinforce the program effectiveness and adapt adequately to the older adults’ needs. Thus, combining multiple disciplines within the same intervention would enhance its impact by simultaneously addressing various dimensions among older adults: *“It will have a better effect because we are acting on all the determinants of deconditioning at the same time.” (OA 1).* Another participant revealed: *“It encompasses all our faculties: the physical, the intellectual, the whole. All these things would keep us vigilant and shining.” (OA 6).* Several positive outcomes were expected by older adults such as enhanced mental well-being, physical function, cognitive abilities and social capabilities. Beyond these anticipations, some participants (*n* = 3/8) pointed to the program potential in fostering health self-management among older adults. Importantly, they believed that *PromoSanté* could be a valuable tool to empower older adults with more knowledge and make them conscious about their health condition, so that they would consider their needs carefully: *“What I like about this program is that it allows me to discover my strengths, weaknesses, and what to do to stop the decline” (OA 5).* The self-management topic was also considered highly important by HCP as it would help older adults to maintain their autonomy and better manage their health. According to them, this kind of multidisciplinary program could be useful in some rehabilitation contexts where patients would be able to regain their abilities and sustain healthy behaviors in the long term and prevent relapses.

Several expected benefits also emerged from interviews with key actors, particularly related to their practices with older adults. The project platform, described by the interviewer, was seen as a valuable tool that would not only help professionals upload and better manage the content of the intervention but also provide older adults with continuous access to this content. For instance, one of the interviewed HCP shared: *“It would be a platform that allows for easy information sharing, the creation of dedicated pages, and the development of a small digital library that users could consult.” (HCP 2).* The digital platform was perceived to be particularly efficient in facilitating the follow-up between healthcare providers and older adults. Indeed, Participants highlighted the platform ability to support synchronous sessions allowing for real-time interactions. Some participants (n = 2/6) also found that the virtual format could reinforce interventions’ quality by enabling more frequent sessions throughout the week. They believed that increased session frequency could enhance the effectiveness of the interventions and improve participants’ engagement. *“I also have a vision of the quality of care because this approach will allow to do four sessions a week instead of just one.” (HCP 2)*.

Moreover, some HCP (*n* = 3/6) highlighted that online interventions could optimize resources’ allocation within healthcare organizations. Indeed, virtual sessions could serve as a viable alternative to avoid home visits and allow for efficient management of human and material resources. A key actor mentioned: *“If the healthcare professional connects with the patient remotely, it would require minimal time and would significantly optimize the number and quality of interventions.” (HCP 2)*. To ensure the expected performance of the program, key actors emphasized the program adaptability to different organizational contexts. According to them, the design should prioritise ease of application and transferability so that the project could be seamlessly integrated into health care practices for older adults.

#### 3.2.2 Effort expectancy

The theme Effort Expectancy, a key construct in the UTAUT2, was identified broadly in verbatims referring to participants’ perceptions regarding the ease of use of the proposed program. Older Adults insisted during interviews on the importance of carefully adapting exercises’ intensity to their physical capacities and keeping gradual progression over the intervention. A flexible and inclusive approach such as including both seated and standing exercises was recommended by older adults to facilitate engagement and long-term adherence to the program. Moreover, older adults voiced that the program should not be overly demanding in terms of time commitment and availability. They expressed that overwhelming schedule could represent a barrier to participation. *“If I was asked to do something that was too demanding in terms of time, no, no, I do not think I would participate.” (OA 3).* Older adults also pointed to the learning efforts they would need to become familiar with using the platform.

Similarly, HCP highlighted the importance of designing a user-friendly interface to ensure the ease of its adoption by older adults. According to them, the platform interface should be simple, intuitive and easily accessible to minimize technical barriers, facilitating seamless navigation and interaction*. “What would help us to perpetuate it and implement it in our community is a platform that would be adapted and easily accessible by older adults.” (HCP 3).* Furthermore, HCP added that future users of *PromoSanté*, whether they are HCP or older adults, should properly position their cameras, especially during physical activity sessions. On the one hand, it is essential that the participant can clearly see the kinesiologist’s movements to follow the exercises accurately. On the other hand, the kinesiologist should be able to monitor the participant’s execution of the movements, ensuring correct form and reducing the risk of injury.

#### 3.2.3 Factors influencing implementation

During the interviews, several factors that could affect the implementation and adoption of *PromoSanté* were mentioned, particularly by HCP, whether as facilitators or barriers (Table 4). Themes that were identified as facilitators include co-design, improved digital literacy and availability of resources. Themes related to barriers were classified into two categories: individual and organizational barriers.

##### 3.2.3.1 Facilitators

Almost all HCP emphasized the necessity of involving key actors, such as HCP and older adults, from the early stages of the design phase. The actors’ perspective would guide the program design in alignment with the essential needs. Accordingly, co-design was considered as a key facilitator in ensuring program accessibility, enhancing its acceptance and fostering its successful implementation. “*It requires a big change in the way professionals work, but if we involve them from the beginning, it’s easier and also gives us a good understanding of their reality*.” *(HCP 4).* Additionally, HCP also noted that since the COVD-19 pandemic, older adults have improved their digital literacy. This increased familiarity with technology tools would facilitate the adoption of *PromoSanté* and maintain its usability. In this context, participants from HCP group (*n* = 3/6) suggested providing a user guide and a training session to familiarize users with the virtual interface: “*Some people will need a user guide: If this happens, here’s how to get out of it, or a bit of training*.” *(HCP 5).*

The availability of technical support was also considered as a crucial factor to ensure that older adults can resolve eventual problems when accessing the platform. Furthermore, accessible and responsive assistance would make *PromoSanté* users feel more confident, thus enhancing their engagement.

In addition, several organizational factors were enumerated by key actors to facilitate the program implementation such as the provision of logistical, financial and human resources. For instance, the development of asynchronous modules requires specialized expertise in video editing to ensure engaging and visually appealing content. Besides, the organization where the program will take place, should provide professionals with an appropriate environment, preferably quiet and free from distractions. Particularly, it was recommended that the kinesiologist and the occupational therapist remain in a quiet room to ensure fluent communication with older adults and avoid disruptions during synchronous sessions.

##### 3.2.3.2 Barriers

###### 3.2.3.2.1 Individual barriers

Some barriers that may impede the effective implementation of *PromoSanté* were also reported. Individually, older adults can experience privacy concerns, age-related challenges, and accessibility issues as well.

HCP (*n* = 2/6) indicated that older adults may have privacy concerns. They mentioned that some participants in previous interventions refused to open their cameras during synchronous sessions.

“We recently conducted a survey on our virtual activities, one of the questions was whether people were comfortable leaving their cameras open, and half the respondents didn’t want to open their cameras.” *(HCP 3).*

Some key actors (*n* = 3/6) also revealed concerns regarding older adults’ ability to adopt the digital platform given that it represents a new technological tool for them. They thought that aging may affect people’s learning curve and pose challenges in using new digital tools.

Besides, accessibility issues to Internet and technological devices may decrease the program feasibility as mentioned by some interviewees (*n* = 2/6). According to their experience, HCP mentioned that connecting to online content often requires a complex authentication procedure, especially when accessing a videoconference session.

###### 3.2.3.2.2 Organizational barriers

Transforming face-to-face interventions into a virtual format can pose certain organizational challenges. On the one hand this transition requires integrating new work practices that could meet resistance from health care professionals. Indeed, they may have concerns about workflow disruptions and the effectiveness of virtual interaction, which could make them reluctant to online programs: “*It’s complicated for them to change practices. You really must show that it’s going to help them in their work, save time and that it’s going to be effective*.” *(HCP 2)*. Another point to consider is that organizations, notably those in the public sector, often have strict standards regarding cybersecurity. Therefore, the program implementation can require prolonged approval process in such settings.

#### 3.2.4 *PromoSanté* development

Participants including HCP and older adults cited advantages for each modality and ultimately recommended a multimodal approach integrating in-person, synchronous and asynchronous sessions. Taken together, this hybrid model would ensure continuous follow-up with health care providers while offering flexibility through virtual options, thereby addressing diverse needs and preferences: “*It has to be hybrid, and that way older adults have a face-to-face and a virtual follow-up*.” *(HCP 5).*

One of HCP suggested starting the program with an in-person session to build an initial contact and a social link between participants. The social dimension asserted a particular interest to both key actors and older adults showing the importance of interactions to enhance older adults’ adherence to physical exercise sessions. Multidisciplinary content was also reported as a common preference across participants. They highlighted how involving mental and social dimensions could reinforce the effectiveness of physical activity interventions by fostering group cohesion. This holistic approach was expected to further improve older adults’ engagement throughout the program by addressing their psychological and social well-being needs: “*It is always valuable to incorporate not only the physical aspect but also mental health and psychosocial support*.” *(OA 8).*

Regarding session frequency, HCP (*n* = 2/6) recommended more than one physical activity session per week to achieve significant health benefits and prevent physical deconditioning among older adults. Virtual modalities would make easier to increase the frequency of sessions while maintaining more flexibility for program users. Besides, the number of older adults per group was also a crucial factor to consider when designing the program implementation. Indeed, one HCP reported that, based on prior experience, physical activity programs were typically conducted in small groups of older adults to create an intimate and supportive environment for them. Accordingly, this preference suggests that smaller groups may build a sense of belonging and foster engagement in the program.

### 3.3 The user journey map of *PromoSanté*

**Figure 1:**
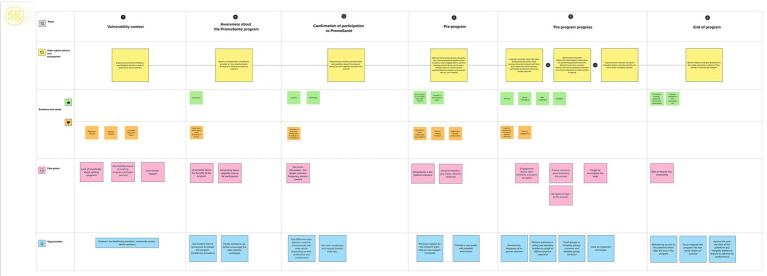
User journey map of *PromoSanté* program.

After interview analysis and co-design discussions with key partners, a user journey map was created to illustrate the expected experience of older adults when participating in *PromoSanté* (Figure 1). Overall, six key phases that represent the main steps older adults will go through from the start to the completion of the program were identified. Each phase is described through 4 parts: (1) actions, (2) emotions and needs, (3) pain and (4) opportunities. More specifically, *part 1-Actions* refers to what the user has to perform at each phase to progress through the experience. This helps identify touchpoints that are the specific interactions where the user encounters the program key actors. *Part 2-Emotions* describes what the older adults are feeling and thinking about in each step and identifies what they need to continue engaging in the program. The *part 3-Pain* helps to predict the problems, frustrations or barriers the users may face at different stages of the journey. The *part 4-Opportunities* focuses on how these challenges (pain points) can be addressed and suggests meaningful solutions to improve the user experience.

#### 3.3.1 Phase1: Vulnerability context

The first phase highlights the situations of vulnerability of older adults as described above. Indeed, limitations can influence several aspects of older adults’ daily lives leading to less physical capacity and social interactions. Several barriers can exacerbate these situations of vulnerability including insufficient awareness of healthy aging programs and accessibility challenges. To address these barriers, the research team of *PromoSanté* should implement effective strategies to promote the program notably by mobilizing healthcare providers within clinical settings, and staff in community center.

#### 3.3.2 Phase 2: Awareness about the *PromoSanté* program

The *PromoSanté* program will be introduced to older adults primarily through trusted referral resources: healthcare providers and the community center network (newsletter, private Facebook group, posters, etc.). They may express interest; however, they may also be uncertain regarding their eligibility for participation. They may also feel doubtful about whether the program can really fulfil their needs, and their capacity to use the virtual platform. At this stage, family members can play an important role by reassuring older adults that they will assist them to overcome technical difficulties when using the platform.

#### 3.3.3 Phase 3: Confirmation of participation

This step is crucial in the user journey as it represents the decision-making point. The older adult makes an initial contact with the research team to obtain more details about the program. At this stage, the research team should clearly explain what participation entails. An empathic communication will help build a trustful link with the participants and make them feel comfortable to ask questions about the program. Older adults will confirm their participation after ensuring that all the eligibility criteria are met.

#### 3.3.4 Phase 4: Pre-program

This phase is established to help older adults get familiar with the use of the digital platform. A first training session will be planned to introduce the platform and show how to access to the program online. Then, a second session will take place to learn how to assist to synchronous sessions and get familiar with the different tools and settings on the videoconference platform such as the microphone and the camera. This step can mitigate older adults’ concerns and raise their confidence in their ability to use the digital tools. However, some older adults, especially those with limited digital literacy, may experience frustration with this new experience. A one-on-one technical support by the research team will be helpful to reduce drop-out risk from the program and make participants feel more supported.

#### 3.3.5 Phase 5: The program process

The program will be carried out using hybrid modalities including in-person, synchronous and asynchronous sessions. In-person sessions will take place at the community center where older adults will have the opportunity to meet each other and build a new social network. Older adults will also assist to physical activity and life redesign sessions animated respectfully by a kinesiologist and an occupational therapist. Furthermore, older adults must accomplish asynchronous modules at their own pace. This content includes comic strips, recorded videos of physical activity, or some readings. Emotions during this phase can vary drastically, between the beginning and the end of the program. As mentioned during qualitative interviews, older adults will need some time to gain familiarity with the platform and integrate the program into their weekly routines. Considering transport and logistic challenges reported by older adults and community center staff, it was decided to include a bi-monthly in-person session to afford more flexibility. Ongoing technical support by the research team can help sustain participants’ engagement. As well, the program will be held in small groups as recommended to protect intimacy and minimize privacy concerns.

#### 3.3.6 Phase 6: The end of program

At the end of the program, older adults may experience physical, mental and social benefits. They may also report increased autonomy and enhanced knowledge about the aging process. Furthermore, the program may help them develop stronger self-management skills to better handle their health conditions. Capturing older adults’ feedback at this phase, and whether they would adopt such a program is extremely useful to inform program refinement and identify opportunities for improvement.

## 4 Discussion

*PromoSanté* is an initiative that integrates physical activity, preventive occupational therapy, and psychological support which aim to promote active lifestyles among older adults to reduce physical and mental deconditioning. Overall, the main finding is that all the participants (older adults and HCP) viewed PromoSanté as a valuable multidisciplinary resource for supporting the health and well-being of older adults. The proposed virtual platform was also well received, as participants, particularly HCP, appreciated the flexibility it offers and its potential to enhance engagement.

The qualitative findings of this study revealed several forms of vulnerability that can be experienced by older adults physically, psychologically and socially. Physical vulnerability, mostly known by frailty, and social vulnerability in older adults are becoming an increasing worldwide issue (55, 56). These two forms of vulnerability were found to be interconnected, as documented in the systematic review by Hanlon et al., indicating that frailty can lead to increased social isolation and loneliness, while social vulnerability can exacerbate frailty progression and hinder recovery (57). Accordingly, it is recommended for future interventions supporting healthy aging strategies, to provide multidisciplinary content that would address different forms of vulnerability in older adults. This aligns with the findings of our study where multidisciplinarity was appreciated by participants and considered as a promising approach to meet older adults’ needs. Using a holistic approach showed more effective results when targeting key dimensions of aging –physical, mental, and social – simultaneously (58). For instance, interventions can involve behavioral or cognitive components alongside exercise training to gain broader benefits and improve overall well-being in older adults. On the other hand, transport difficulties were outlined in our study among major barriers to healthy aging. This further emphasized the need for virtual modalities to facilitate older adults access to the available programs and mitigate transportation constraints (59). By minimizing logistical barriers, virtual programs may be relevant to enhance consistent participation, reduce missed sessions, and support sustained older adults’ engagement in healthy aging programs over time. Overall, the barriers mentioned above can further support the proposed format of the PromoSanté program as a multidisciplinary program that deploys virtual modalities in order to address physical and social deconditioning in older adults.

Participants’ perspectives on the *PromoSanté* program were grouped into four themes: (1) performance expectancy, (2) effort expectancy, (3) factors influencing implementation, and (4) design preferences. Performance expectancy, effort expectancy, and facilitating conditions emerged as key elements shaping how participants viewed the program’s usefulness and usability. This aligns with Baik et al. (60), who applied these three UTAUT2 dimensions—along with age as a moderating factor—to explore how older adults and professionals perceive the integration of technological tools to enhance motivation for physical activity. Their findings support the relevance of UTAUT2 as a predictive framework for understanding end-users’ intentions to adopt digital health interventions. In our study, performance expectancy was the most frequently mentioned theme, suggesting that perceived benefits of the program may strongly influence engagement and participation.

These findings are consistent with previous research showing that retention in physical activity programs often depends on whether older adults perceive these interventions as enhancing their health or quality of life (60–62). In our study, participants highlighted some expected benefits such as improved physical and mental well-being. They also felt that integrating components of the *Life Redesign* program could strengthen engagement in *PromoSanté* by increasing awareness of age-related challenges and encouraging social participation. Although multidisciplinary interventions have been shown to support psychological well-being and physical activity among older adults (63, 64), such programs remain relatively uncommon, partly because they require coordination across multiple professional domains—an effort that can introduce logistical and communication challenges. Participants also noted that the digital format of *PromoSanté* could help address accessibility barriers, a perspective echoed in earlier studies showing that virtual programs are particularly valuable for individuals facing transportation difficulties (61, 65, 66).

Within the theme of effort expectancy, participants stressed the importance of a user-friendly interface that is easy to navigate, ensuring that participation in the program does not become burdensome—particularly for older adults with limited technological experience. The notion of usability is well documented in the literature, emphasizing principles such as simple navigation and a clear, intuitive layout (67, 68). Participants also noted that virtual sessions could reduce both physical effort and mental strain by offering greater flexibility. Traveling by car or public transit can be tiring and stressful for many older adults, whereas technology allows them to take part in sessions from home. This increased flexibility aligns with previous studies showing that online interventions can save time, reduce fatigue, and lower costs for participants (69–71).

Participants identified several facilitators, with co-design emerging as a particularly valuable approach. They strongly recommended involving users and key actors early in the development process to ensure that diverse perspectives are considered and that the program aligns with users’ real needs. This is consistent with literature showing that co-design enhances the usability, acceptability, and long-term sustainability of digital physical activity programs for older adults (72–75). Older adults in this study expressed interest in using digital tools to support active lifestyles, and key actors noted that digital literacy among this population has improved since the COVID-19 pandemic, which accelerated technology adoption. These findings align with previous research indicating that older adults are increasingly motivated and confident in using digital platforms (60, 66, 76).

Participants also suggested that training resources—such as introductory workshops or user guides—would help facilitate the use of *PromoSanté*, echoing Garcia et al. (77), who recommended providing technology training before virtual interventions. Finally, HCP emphasized that successful implementation will require organizational investments. This reflects findings by Dawson et al. (78), who noted that telehealth adoption depends on sustained institutional commitment, including staff training and technical support, to ensure effective uptake and long-term viability.

Regarding barriers, HCP raised concerns about privacy and confidentiality, echoing prior research showing that some individuals avoid turning on their cameras due to discomfort, which can limit engagement and the quality of feedback (60, 79, 80). Our findings also align with Islam et al. (81), who reported that older adults face accessibility issues, usability challenges, limited digital literacy, and privacy worries when engaging in online interventions. That study further noted that virtual programs may reduce opportunities for social interaction compared to in-person activities, potentially impacting motivation and participation. Despite these well-documented challenges associated with online physical activity programs, institutional barriers—such as organizational readiness, staff support, or resource constraints—remain largely underexplored in the current literature.

In this study, both older adults and HCP favored a hybrid format combining virtual and in-person sessions. This mirrors prior findings showing that hybrid models offer the flexibility of online participation while preserving the social benefits of face-to-face classes (66, 82). Participants also valued using both synchronous and asynchronous formats to support engagement. Peng et al. (83) similarly noted that synchronous sessions provide real-time interaction and feedback, whereas asynchronous content offers greater flexibility, recommending a combination of both. Other studies report that synchronous classes enhance participation, social connection, and supervision compared to asynchronous modes (76). Regarding frequency, one to three weekly synchronous sessions appears feasible and acceptable for community-dwelling older adults and may help increase physical activity levels (36).

### 4.1 User journey and implementation perspectives

Based on the aforementioned results and co-design meetings, the PromoSanté program will include in-person physical exercise and Lifestyle Redesign sessions performed on the same day at the community center in Sherbrooke city, once every 2 weeks. Virtual sessions will take place weekly through the platform and will include synchronous physical exercises sessions animated by a kinesiologist, and live exchanges sessions of Lifestyle Redesign animated by an occupational therapist. Besides synchronous sessions, the program will include asynchronous modules in the form of prerecorded videos of physical exercises, reflexive activities about healthy aging and psychological support delivered in comic strips. Technical support will be provided by the research team for both HCP and older adults when needed. Through participating to the implementation of the PromoSanté program, HCP can gain more advanced competencies in delivering virtual interventions among older adults, and increasing practices efficiency and flexibility.

### 4.2 Study strengths and limitations

This qualitative study brings together perspectives from older adults as well as HCP from clinical and community settings, offering a rich and multidimensional view of the factors that influence healthy aging. By engaging diverse user profiles, the study provides valuable insights for designing aging-friendly programs that are better aligned with real-life needs and more likely to achieve long-term adoption. The UTAUT2 model served as a guiding framework for data collection and analysis, helping to explore participants’ perceptions and predict behavioral intentions toward adopting *PromoSanté.* While UTAUT2 proved useful, the analysis also uncovered additional themes—particularly related to organizational and contextual factors—that extend beyond the model’s scope. As highlighted by Williams et al. (84), future research should therefore consider both individual and institutional determinants when designing and implementing digital health interventions.

This study also has limitations. The sample was predominately female, which can limit the generalizability of the findings due to possible gender related differences in health behaviors, and preferences for technology use. The female overrepresentation particularly in north American studies (85), may be partly explained by gendered patterns of social participation and health-related engagement observed in later life. Previous work conducted by Mehranfar et al. (86) emphasized that women often face greater constraints due to gender roles and inequities, which may further increase their interest in interventions designed to promote health, well-being, and social engagement.

The sample size of 14 participants may not fully capture the diversity of experiences within an aging population with varied needs. Moreover, all participants were recruited from an urban area, which may affect the transferability of results to regions with different levels of digital literacy, and technical infrastructure availability. As such, certain contextual barriers and facilitators associated with rural, and resource-limited settings may be underrepresented. However, this study did not seek exhaustive thematic coverage of older adults and HCP perspectives, but rather to gather actionable and context-specific insights to support the co-construction of the PromoSanté program. Considering that the intended program will be implemented in Sherbrooke city as a first implementation initiative, it is important to consider local and contextual settings. Nevertheless, several findings retain conceptual transferability and can orientate the design and implementation of similar healthy aging programs. For instance, the reported challenges, expectations and preferences could be applicable to different contexts of implementation. Contextual disparities specifically in organizational resources and participants’ digital literacy may influence how these findings are translated into practice. Finally, the findings reflect the views of individuals who were willing to participate, which may differ from those who chose not to engage and who may hold different perspectives.

## 5 Conclusion

This study provided exploratory insights into several challenges that may hinder healthy aging and increase vulnerability risk among older adults. Transport limitations were commonly described by participants as an important obstacle, decreasing the ability to participate in activities that support physical, cognitive, and social well-being. Against this backdrop, HCP perceived the digital platform proposed for delivering *PromoSanté*’s virtual content as an innovative solution, providing insights into potential tools that may enhance engagement and continuity of care. Older adults also expressed interest in the program’s multidisciplinary design, perceiving its potential to address physical, psychological, and social needs. Additionally, the study offers exploratory insights and formative observations that may inform the development of future similar active-aging interventions. Taken together, these findings highlight the value of involving future users early in the conception of telehealth programs for healthy aging. Consulting older adults and HCP from the outset helps ensure that emerging interventions are adequately aligned with the real-world conditions in which they will be implemented.

## Data Availability

The datasets presented in this article are not readily available because they consist of verbatim interview transcripts. Requests to access the datasets should be directed to patrick.boissy@usherbrooke.ca.

## References

[1] Siebens H. Mobility disorders in older adults: the role of deconditioning. J Back Musculoskelet Rehabil. (1994) 4:91–6. doi: 10.3233/BMR-1994-4206, 24572020

[2] Wang DX Yao J Zirek Y Reijnierse EM Maier AB. Muscle mass, strength, and physical performance predicting activities of daily living: a meta-analysis. J Cachexia Sarcopenia Muscle. (2020) 11:3–25. doi: 10.1002/jcsm.12502, 31788969 PMC7015244

[3] Visser M Sääksjärvi K Burchell GL Schaap LA. The association between muscle mass and change in physical functioning in older adults: a systematic review and meta-analysis of prospective studies. Eur Geriatr Med. (2025) 16:1731–48. doi: 10.1007/s41999-025-01230-y 40407980 PMC12528211

[4] Blodgett J Theou O Kirkland S Andreou P Rockwood K. The association between sedentary behaviour, moderate-vigorous physical activity and frailty in NHANES cohorts. Maturitas. (2015) 80:187–91. doi: 10.1016/j.maturitas.2014.11.010, 25542406

[5] Harvey JA Chastin SF Skelton DA. Prevalence of sedentary behavior in older adults: a systematic review. Int J Environ Res Public Health. (2013) 10:6645–61. doi: 10.3390/ijerph10126645, 24317382 PMC3881132

[6] Cunningham C O’Sullivan R Caserotti P Tully MA. Consequences of physical inactivity in older adults: a systematic review of reviews and meta-analyses. Scand J Med Sci Sports. (2020) 30:816–27. doi: 10.1111/sms.13616, 32020713

[7] Leung PM Ejupi A van Schooten KS Aziz O Feldman F Mackey DC . Association between sedentary behaviour and physical, cognitive, and psychosocial status among older adults in assisted living. Biomed Res Int. (2017) 2017:9160504. doi: 10.1155/2017/9160504 28913360 PMC5587924

[8] Chu A Lu Y Zhang H Jiang Y. Sedentary behavior, physical activity, social participation, and loneliness among community-dwelling older adults in China. J Aging Phys Act. (2023) 31:987–94. doi: 10.1123/japa.2022-0205, 37442551

[9] Gyasi RM Phillips DR Asante F Boateng S. Physical activity and predictors of loneliness in community-dwelling older adults: the role of social connectedness. Geriatr Nurs. (2021) 42:592–8. doi: 10.1016/j.gerinurse.2020.11.004 33246663

[10] Yasunaga A Koohsari M Shibata A Ishii K Miyawaki R Araki K . Sedentary behavior and happiness: the mediation effects of social capital. Innov Aging. (2021) 5:igab044. doi: 10.1093/geroni/igab044, 34859156 PMC8633129

[11] Schrempft S Jackowska M Hamer M Steptoe A. Associations between social isolation, loneliness, and objective physical activity in older men and women. BMC Public Health. (2019) 19:74. doi: 10.1186/s12889-019-6424-y, 30651092 PMC6335852

[12] Institut de la statistique du Québec. La population du Québec d’ici 2066: une croissance qui se poursuit, mais qui ralentit. (2019); Available online at: https://www.quebec.ca/nouvelles/actualites/details/la-population-du-quebec-dici-2066-une-croissance-qui-se-poursuit-mais-qui-ralentit?utm_source=chatgpt.com (Accessed April 04, 2026).

[13] Statistics Canada. Population estimates on July 1st, by age and sex. (2023); Available online at: https://www150.statcan.gc.ca/t1/tbl1/en/tv.action?pid=1710000501&request_locale=en (Accessed April 04, 2026).

[14] Izquierdo M Duque G Morley JE. Physical activity guidelines for older people: knowledge gaps and future directions. Lancet Healthy Longev. (2021) 2:e380–3. doi: 10.1016/S2666-7568(21)00079-9, 36098146

[15] Chan YY Lim KK Omar MA Mohd Yusoff MF Sooryanarayana R Ahmad NA . Prevalence and factors associated with physical inactivity among older adults in Malaysia: a cross-sectional study. Geriatr Gerontol Int. (2020) 20:49–56. doi: 10.1111/ggi.13977, 33370865

[16] Collado-Mateo D Lavín-Pérez AM Peñacoba C Del Coso J Leyton-Román M Luque-Casado A . Key factors associated with adherence to physical exercise in patients with chronic diseases and older adults: an umbrella review. Int J Environ Res Public Health. (2021) 18:2023. doi: 10.3390/ijerph18042023 33669679 PMC7922504

[17] Choi NG DiNitto DM Marti CN Choi BY. Telehealth use among older adults during COVID-19: associations with sociodemographic and health characteristics, technology device ownership, and technology learning. J Appl Gerontol. (2022) 41:600–9. doi: 10.1177/07334648211047347, 34608821 PMC8847316

[18] Khanassov V Ilali M Ruiz AS Rojas-Rozo L Sourial R. Telemedicine in primary care of older adults: a qualitative study. BMC Prim Care. (2024) 25:259. doi: 10.1186/s12875-024-02518-x, 39020277 PMC11253566

[19] World Health Organization International Telecommunication Union. WHO-ITU Global Standard for Accessibility of Telehealth Services. Geneva: World Health Organization (2022).

[20] World Health Organization. Consolidated Telemedicine Implementation Guide. 1st ed. Geneva: World Health Organization (2022). p. 1.

[21] Garrido ND Reis VM Vilaça-Alves JM Lucas GC Godinho IL Peixoto R . Impact of tele-exercise on quality of life, physical fitness, functional capacity and strength in different adult populations: a systematic review of clinical trials. Front Sports Act Living. (2025) 7:1505826. doi: 10.3389/fspor.2025.1505826, 39949715 PMC11821610

[22] Brown RC Coombes JS Rodriguez KJ Hickman IJ Keating SE. Effectiveness of exercise via telehealth for chronic disease: a systematic review and meta-analysis of exercise interventions delivered via videoconferencing. Br J Sports Med. (2022) 56:1042–52. doi: 10.1136/bjsports-2021-105118 35715175

[23] Pizarro-Mena R Duran-Aguero S Causa-Vera M Rios-Duran C Parra-Soto S. Perceived facilitators and barriers, from the perspective of users, of a multicomponent intervention in older people using an asynchronous telehealth modality during the COVID-19 pandemic: a qualitative research. J Aging Res. (2025) 2025:6839569. doi: 10.1155/jare/6839569 40200977 PMC11976052

[24] Académie de la transformation numérique. Les aînés connectés au Québec. (2022) Québec: Académie de la transformation numérique.

[25] Choi NG DiNitto DM. The digital divide among low-income homebound older adults: internet use patterns, eHealth literacy, and attitudes toward computer/internet use. J Med Internet Res. (2013) 15:e93. doi: 10.2196/jmir.2645, 23639979 PMC3650931

[26] Cohen-Mansfield J Muff A Meschiany G Lev-Ari S. Adequacy of web-based activities as a substitute for in-person activities for older persons during the COVID-19 pandemic: survey study. J Med Internet Res. (2021) 23:e25848. doi: 10.2196/25848, 33439851 PMC7836908

[27] Istepanian RSH. Mobile health (m-health) in retrospect: the known unknowns. Int J Environ Res Public Health. (2022) 19:3747. doi: 10.3390/ijerph19073747 35409431 PMC8998037

[28] Alruwaili MM Shaban M Elsayed Ramadan OM. Digital health interventions for promoting healthy aging: a systematic review of adoption patterns, efficacy, and user experience. Sustainability. (2023) 15:16503. doi: 10.3390/su152316503

[29] Wilson J Heinsch M Betts D Booth D Kay-Lambkin F. Barriers and facilitators to the use of e-health by older adults: a scoping review. BMC Public Health. (2021) 21:1–12. doi: 10.1186/s12889-021-11623-w 34399716 PMC8369710

[30] Walha R Koubaa N Chagnon M Lortie-Milner E Aubertin-Leheudre M Levasseur M . E-health interventions for promoting physical activity in aging adults: a scoping review. Telemed E-Health. (2025) 31:531–9. doi: 10.1089/tmj.2024.0414 39757866

[31] Dawson R Oliveira JS Kwok WS Bratland M Rajendran IM Srinivasan A . Exercise interventions delivered through telehealth to improve physical functioning for older adults with frailty, cognitive, or mobility disability: a systematic review and meta-analysis. Telemed E-Health. (2024) 30:940–50. doi: 10.1089/tmj.2023.0177, 37975811 PMC11035924

[32] Beauchamp MR Hulteen RM Ruissen GR Liu Y Rhodes RE Wierts CM . Online-delivered group and personal exercise programs to support low active older adults’ mental health during the COVID-19 pandemic: randomized controlled trial. J Med Internet Res. (2021) 23:e30709. doi: 10.2196/30709, 34328433 PMC8330630

[33] Yerlikaya T Öniz A Özgùren M. The effect of an interactive tele rehabilitation program on balance in older individuals. Neurol Sci Neurophysiol. (2021) 38:180–6. doi: 10.4103/nsn.nsn_91_21

[34] Granet J Peyrusqué E Ruiz F Buckinx F Abdelkader LB Dang-Vu T . Online physical exercise intervention in older adults during lockdown: can we improve the recipe? Aging Clin Exp Res. (2023) 35:551–60. doi: 10.1007/s40520-022-02329-z 36635450 PMC9838396

[35] Granet J Peyrusqué E Ruiz F Buckinx F Abdelkader LB Dang-Vu TT . Web-based physical activity interventions are feasible and beneficial solutions to prevent physical and mental health declines in community-dwelling older adults during isolation periods. J Gerontol A Biol Sci Med Sci. (2023) 78:535–44. doi: 10.1093/gerona/glac127, 35675174 PMC9384240

[36] Diaz MFF Leadbetter B Pitre V Nowell S Sénéchal M Bouchard DR. Synchronous group-based online exercise programs for older adults living in the community: a scoping review. J Aging Phys Act. (2024) 32:703–17. doi: 10.1123/japa.2023-0214, 38823794

[37] Lévesque MH Trépanier J Sirois MJ Levasseur M. Effects of lifestyle redesign on older adults: a systematic review. Can J Occup Ther. (2019) 86:48–60. doi: 10.1177/0008417419830429 30884959

[38] De Coninck L Declercq A Bouckaert L Döpp C Graff MJL Aertgeerts B. Promoting meaningful activities by occupational therapy in elderly care in Belgium: the ProMOTE intervention. BMC Geriatr. (2024) 24:275. doi: 10.1186/s12877-024-04797-6, 38509458 PMC10953191

[39] Uceda-Portillo C Aranda-Valero S Moruno-Miralles P. Occupational therapy interventions to improve the quality of life of older adults with dementia living in nursing homes: a systematic review. Healthcare. (2024) 12:896. doi: 10.3390/healthcare12090896 38727453 PMC11083416

[40] Chagnon M Levasseur M Boissy P. Telehealth interventions in occupational therapy with older adults: results from a scoping review targeting better health promotion. Aust Occup Ther J. (2024) 71:190–208. doi: 10.1111/1440-1630.12910, 37885381

[41] Taylor DM Cameron JI Walsh L McEwen S Kagan A Streiner DL . Exploring the feasibility of videoconference delivery of a self-management program to rural participants with stroke. Telemed J E Health. (2009) 15:646–54. doi: 10.1089/tmj.2008.0165, 19694589

[42] Sakakibara BM Lear SA Barr SI Goldsmith CH Schneeberg A Silverberg ND . Telehealth coaching to improve self-management for secondary prevention after stroke: a randomized controlled trial of stroke coach. Int J Stroke. (2022) 17:455–64. doi: 10.1177/17474930211017699, 33949270

[43] Hossain M Leminen S Westerlund M. A systematic review of living lab literature. J Clean Prod. (2019) 213:976–88. doi: 10.1016/j.jclepro.2018.12.257

[44] Lévesque MH Trépanier J Tardif MÈ Lalanne K Boudriau M Ainsley S . Remodeler sa vie®(Lifestyle Redesign): première étude pilote auprès d’aînés franco-canadiens. Can J Occup Ther. (2020) 87:241–52. doi: 10.1177/0008417420929508 32580571

[45] move50+. The move 50+ program [internet]. (2017). Available online at: https://move50plus.ca/ (Accessed January 20, 2026).

[46] Blandford A Gibbs J Newhouse N Perski O Singh A Murray E. Seven lessons for interdisciplinary research on interactive digital health interventions. Digit Health. (2018) 4:2055207618770325. doi: 10.1177/2055207618770325 29942629 PMC6016567

[47] Greenhalgh T Shaw S Wherton J Hughes G Lynch J C A’C . SCALS: a fourth-generation study of assisted living technologies in their organisational, social, political and policy context. BMJ Open. (2016) 6:e010208. doi: 10.1136/bmjopen-2015-010208, 26880671 PMC4762149

[48] Göttgens I Oertelt-Prigione S. The application of human-centered design approaches in health research and innovation: a narrative review of current practices. JMIR Mhealth Uhealth. (2021) 9:e28102. doi: 10.2196/28102, 34874893 PMC8691403

[49] Tamilmani K Rana NP Wamba SF Dwivedi R. The extended unified theory of acceptance and use of technology (UTAUT2): a systematic literature review and theory evaluation. Int J Inf Manag. (2021) 57:102269. doi: 10.1016/j.ijinfomgt.2020.102269

[50] Kavandi H Jaana M. Factors that affect health information technology adoption by seniors: a systematic review. Health Soc Care Community. (2020) 28:1827–42. doi: 10.1111/hsc.13011, 32378769

[51] Doyle L McCabe C Keogh B Brady A McCann M. An overview of the qualitative descriptive design within nursing research. J Res Nurs. (2020) 25:443–55. doi: 10.1177/1744987119880234, 34394658 PMC7932381

[52] Sandelowski M. Whatever happened to qualitative description? Res Nurs Health. (2000) 23:334–40. doi: 10.1002/1098-240X(200008)23:4<334::AID-NUR9>3.0.CO;2-G, 10940958

[53] Bradshaw C Atkinson S Doody O. Employing a qualitative description approach in health care research. Glob Qual Nurs Res. (2017) 4:2333393617742282. doi: 10.1177/2333393617742282 29204457 PMC5703087

[54] Miles H Huberman AM Saldana J. Qualitative data Analysis: A Methods Sourcebook. New York, NY: Sage Publ Inc. (2014). p. 69–104.

[55] O’Caoimh R Sezgin D O’Donovan MR Molloy DW Clegg A Rockwood K . Prevalence of frailty in 62 countries across the world: a systematic review and meta-analysis of population-level studies. Age Ageing. (2021) 50:96–104. doi: 10.1093/ageing/afaa219, 33068107

[56] Surkalim DL Luo M Eres R Gebel K Van Buskirk J Bauman A . The prevalence of loneliness across 113 countries: systematic review and meta-analysis. BMJ. (2022) 376:e067068. doi: 10.1136/bmj-2021-067068, 35140066 PMC8826180

[57] Hanlon P Wightman H Politis M Kirkpatrick S Jones C Andrew MK . The relationship between frailty and social vulnerability: a systematic review. Lancet Healthy Longev. (2024) 5:e214–26. doi: 10.1016/S2666-7568(23)00263-5, 38432249

[58] Lucifora C Villar E. Multi-dimensional healthy aging interventions: evidence from an age-friendly community program in Italy. Ageing Int. (2024) 49:749–71. doi: 10.1007/s12126-024-09567-8

[59] Bella G Borowski E Stathopoulos A. Examining healthcare access with physical vs. telehealth options: promise and peril for socially vulnerable older adults. J Transp Health. (2025) 40:101940. doi: 10.1016/j.jth.2024.101940

[60] Baik D Reeder B Coats H Baker C Jankowski C. Perceptions and attitudes toward a proposed digital health physical activity program among older family caregivers of persons with heart failure: a qualitative study. Inform Health Soc Care. (2023) 48:239–51. doi: 10.1080/17538157.2023.2227704, 37417465 PMC10990475

[61] Ehn M Johansson AC Revenäs Å. Technology-based motivation support for seniors’ physical activity—a qualitative study on seniors’ and health care professionals’ views. Int J Environ Res Public Health. (2019) 16:2418. doi: 10.3390/ijerph16132418, 31288398 PMC6651538

[62] Ratz T Voelcker-Rehage C Pischke CR Muellmann S Peters M Lippke S. Health-related lifestyle and dropout from a web-based physical activity intervention trial in older adults: a latent profile analysis. Health Psychol. (2021) 40:481. doi: 10.1037/hea0001091, 34472906

[63] Shim M Kavanaugh M Lacson C Goldstein-Levitas N Chang H Zhang F . Connected through movement: a feasibility study of online mindfulness-based dance/movement therapy for older adults with age-related cognitive decline during COVID-19. Aging Ment Health. (2024) 28:1676–85. doi: 10.1080/13607863.2024.2364754, 38910361

[64] Zhang Z Jin L Liu J Liao D Zhang X. The impact of social participation on the health status of the older adult: an empirical study based on CGSS 2021 data. PLoS One. (2024) 19:e0305820. doi: 10.1371/journal.pone.0305820, 38917146 PMC11198831

[65] Dagenais M Krajnovic A Galway S Gammage K. Instructors’ perceptions and experiences of teaching online exercise classes to older adults: a qualitative study. J Aging Phys Act. (2023) 32:124–37. doi: 10.1123/japa.2022-0434, 37883633

[66] Taveira F Barbosa B. Older adults’ continuance intentions for online physical exercise classes. Behav Sci. (2024) 14:393. doi: 10.3390/bs14050393, 38785884 PMC11118063

[67] Ritchie S Lawrence V Jones J Corbett A. Engaging older adults in an online physical activity programme to improve cognition: a qualitative study. Int J Geriatr Psychiatry. (2021) 36:1942–9. doi: 10.1002/gps.5617, 34410017

[68] Alley SJ Samra P Rebar AL Schoeppe S Parkinson L Power D . A focus group study of older adults’ perceptions and preferences towards web-based physical activity interventions. Inform Health Soc Care. (2020) 45:273–81. doi: 10.1080/17538157.2019.1656210, 31690152

[69] Akinrolie O Ripat J Strachan S Webber SC Barclay R. Virtual motivational interviewing (VIMINT) to support physical activity: experiences of older adults and counsellors. J Health Psychol. (2024) 29:1416–30. doi: 10.1177/13591053241235094, 38414103 PMC11528923

[70] Gravesande J Almeida de Oliveira L Malik N Vrkljan B Zheng R Gardner PM . Feasibility, usability, and acceptability of online mind–body exercise programs for older adults: a scoping review. J Integr Complement Med. (2023) 29:538–49. doi: 10.1089/jicm.2022.0822, 36944159

[71] Gray SM Franke T Sims-Gould J McKay HA. Rapidly adapting an effective health promoting intervention for older adults—choose to move—for virtual delivery during the COVID-19 pandemic. BMC Public Health. (2022) 22:1172. doi: 10.1186/s12889-022-13547-5, 35690744 PMC9188419

[72] Cairns A Rodda D Wymarra F Bird K. Healthy ageing in remote Cape York: a co-designed integrated allied health service model. Aust J Prim Health. (2024) 30:PY23135. doi: 10.1071/PY23135 38237265

[73] Villa-García L Davey V Peréz LM Soto-Bagaria L Risco E Díaz P . Co-designing implementation strategies to promote remote physical activity programs in frail older community-dwellers. Front Public Health. (2023) 11:1062843. doi: 10.3389/fpubh.2023.1062843, 36960372 PMC10028273

[74] Guell C Panter J Griffin S Ogilvie D. Towards co-designing active ageing strategies: a qualitative study to develop a meaningful physical activity typology for later life. Health Expect. (2018) 21:919–26. doi: 10.1111/hex.12686, 29624803 PMC6186535

[75] Backåberg S Strandberg S Freeman G Katz L Rafiei Milajerdi H Wylant B . Facilitating co-design among older adults in a digital setting: methodological challenges and opportunities. CoDesign. (2025) 21:118–35. doi: 10.1080/15710882.2024.2372595

[76] Mehrabi S Drisdelle S Dutt HR Middleton LE. “If i want to be able to keep going, i must be active.” exploring older adults’ perspectives of remote physical activity supports: a mixed-methods study. Front Public Health. (2024) 12:1328492. doi: 10.3389/fpubh.2024.1328492 38327585 PMC10847274

[77] Garcia LV Davey V Mónica Pérez L Soto L Risco E Carrion C . + ÀGILvirtual: co-design of a remote digital intervention to provide physical exercise within a multi-component program to delay disability in older adults. Int J Integr Care. (2022) 22:223. doi: 10.5334/ijic.icic22109

[78] Dawson R Gilchrist H Pinheiro M Nelson K Bowes N Sherrington C . Experiences of older adults, physiotherapists, and aged care staff in the TOP UP telephysiotherapy program: interview study of the TOP UP interventions. JMIR Aging. (2024) 7:e53010. doi: 10.2196/53010, 38324369 PMC10882472

[79] Peng R Cao Z Hu S Liu X Guo Y Li X . Frail older adults’ needs and preferences for mobile health exercise interventions guided by nudge theory: aqualitative analysis. J Clin Nurs. (2024) 34:1830–9. doi: 10.1111/jocn.17419, 39215431

[80] LeBaron V. Challenges and opportunities in designing and deploying remote health monitoring technology for older adults with cancer. Innov Aging. (2022) 6:igac057. doi: 10.1093/geroni/igac057, 36452048 PMC9701055

[81] Islam MS Frazier MC Harden SM Lim S. Barriers and benefits of online group exercise programs for older adults. J Appl Gerontol. (2024) 43:1397–407. doi: 10.1177/07334648241240599, 38536888

[82] Gerhardsson KM Hassan M Tornberg ÅB Schmidt SM. Usability and feasibility of an online intervention for older adults to support changes to routines and the home ('light, activity and sleep in my daily life’). BMC Public Health. (2024) 24:2808. doi: 10.1186/s12889-024-20309-y, 39402489 PMC11475629

[83] Peng R Chang J Du Y Zhang C Li X Guo Y . Older adults’ perceptions and experiences of engaging in web-and mobile-based physical activity interventions: a systematic review and qualitative meta-synthesis. Geriatr Nurs. (2024) 59:630–8. doi: 10.1016/j.gerinurse.2024.08.025, 39197354

[84] Williams MD Rana NP Dwivedi YK. The unified theory of acceptance and use of technology (UTAUT): a literature review. J Enterp Inf Manag. (2015) 28:443–88. doi: 10.1108/jeim-09-2014-0088

[85] Polit DF Beck CT. International gender bias in nursing research, 2005–2006: a quantitative content analysis. Int J Nurs Stud. (2009) 46:1102–10. doi: 10.1016/j.ijnurstu.2009.02.002, 19268940

[86] Mehranfar S Ceolin G Madani Civi R Keller H Murphy RA Cohen TR . Gender, adverse changes in social engagement and risk of unhealthy eating: a prospective cohort study of the Canadian longitudinal study on aging (2011–2021). Nutrients. (2025) 17:1005. doi: 10.3390/nu17061005 40290021 PMC11946033

